# Comprehensive Analysis of Garcin Syndrome: A Systematic Review of the Etiology, Diagnosis, and Treatment

**DOI:** 10.7759/cureus.70197

**Published:** 2024-09-25

**Authors:** Ali Alshalchy, Rania H Al-Taie, Sajjad G Al-Badri, Mohammed A Bani Saad, Ali A Bani-Saad, Mustafa Ismail

**Affiliations:** 1 Department of Surgery, College of Medicine, University of Baghdad, Baghdad, IRQ; 2 Department of Surgery, College of Medicine, University of Mustansiriyah, Baghdad, IRQ; 3 Department of Surgery, Baghdad Teaching Hospital, Medical City Complex, Baghdad, IRQ

**Keywords:** cranial nerve palsy, diagnosis, garcin syndrome, metastatic disease, treatment

## Abstract

Garcin syndrome is a rare neurological condition characterized by progressive unilateral involvement of multiple cranial nerves, without typical intracranial hypertension. It is often linked with aggressive malignancies and invasive infections; hence, it presents significant diagnostic and therapeutic challenges. Despite the advances in medical technology, the prognosis still remains poor, and there is limited literature on comprehensive reviews regarding its etiology, diagnosis, and management. A Preferred Reporting Items for Systematic Reviews and Meta-Analyses (PRISMA)-based systematic review was performed in order to compile updated evidence related to Garcin syndrome. The PubMed and Scopus databases were comprehensively searched using the search terms "Garcin syndrome" AND ("etiology" OR "diagnosis" OR "treatment" OR "management"). Information was obtained from case reports and focused on common etiologies, clinical presentations, diagnostic methodologies, treatment protocols, and outcomes.

The review discussed very diverse etiologies of Garcin syndrome. The most common among these were skull base tumors, metastatic lesions, and invasive infections like mucormycosis. Most of these were multiple cranial nerve (CN) involvements in which CN V (trigeminal nerve), CN VII (facial nerve), or CN XII (hypoglossal nerve) involvement was common. Advanced imaging, especially MRI, played a very crucial role in diagnosis, showing the presence of extensive bony destruction with the involvement of cranial nerves. Treatment was varied according to the etiology, ranging from chemotherapy and radiotherapy in neoplastic cases to active surgical intervention supported by antifungal therapy in infected cases. Garcin syndrome is a clinical challenge due to its diverse etiologies and complex management profile. While early diagnosis and intervention are emphasized, the prognosis remains grave, especially in cases presenting with metastatic disease or immunocompromised states. Future research should focus on better, more sensitive diagnostic modalities and the investigation of newer therapeutic approaches for Garcin syndrome patients.

## Introduction and background

Garcin syndrome is an infrequent but important neurological disorder, with contemporary or sequential unilateral involvement of multiple cranial nerves, evolving into paralysis without associated disturbance of sensory and motor long tracts or intracranial hypertension. First described by Raymond Garcin in 1926, this syndrome is also known as unilateral global cranial nerve palsy and has been reported with osteoclastic involvement at the skull base, associated with a myriad of underlying pathologies, predominant among which are malignant neoplasms and invasive infections [1]. The causes of Garcin syndrome are diverse, but the majority are related to malignancies, particularly those invading the skull base. These include nasopharyngeal carcinoma, parapharyngeal space tumors, and metastases from distant primary sites such as the breast, lung, and prostate. Interestingly, Garcin syndrome may present initially with metastasis, as illustrated in cases of prostate cancer metastasis to the skull base with resultant extensive cranial nerve involvement [2].

Symptoms of Garcin syndrome usually show an insidious onset, developing with the involvement of one or two cranial nerves and showing a progressive increase in the involvement of other nerves in the advanced stage of the disease. The most common cranial nerves to be involved are the oculomotor, facial, hypoglossal, and others. In spite of such extensive nerve involvement, patients usually do not present with features of raised intracranial pressure or other evidence of central nervous system involvement, which further obscures the diagnosis [3]. Imaging studies, especially MRI, play a very important factor in the diagnosis of Garcin syndrome by detecting lesions at the skull base and determining the extent of involvement of cranial nerves. Skull base involvement due to metastatic tumors may sometimes cause significant morbidity because several cranial nerves may get involved, manifesting in profound neurological deficits [2]. Despite the advances in diagnosis and therapeutic options, the prognosis remains poor for patients with Garcin syndrome because the basic conditions mostly have an aggressive course. Treatment is often symptomatic and palliative to alleviate symptoms and retard the disease process; the response to treatment is often limited, with survival rates still low [1].

The aim, therefore, is to conduct a systematized review of the literature on Garcin syndrome, with an emphasis on the review of the literature concerning etiological factors, diagnostic approaches, and treatment outcomes.

## Review

Method

**Figure 1:**
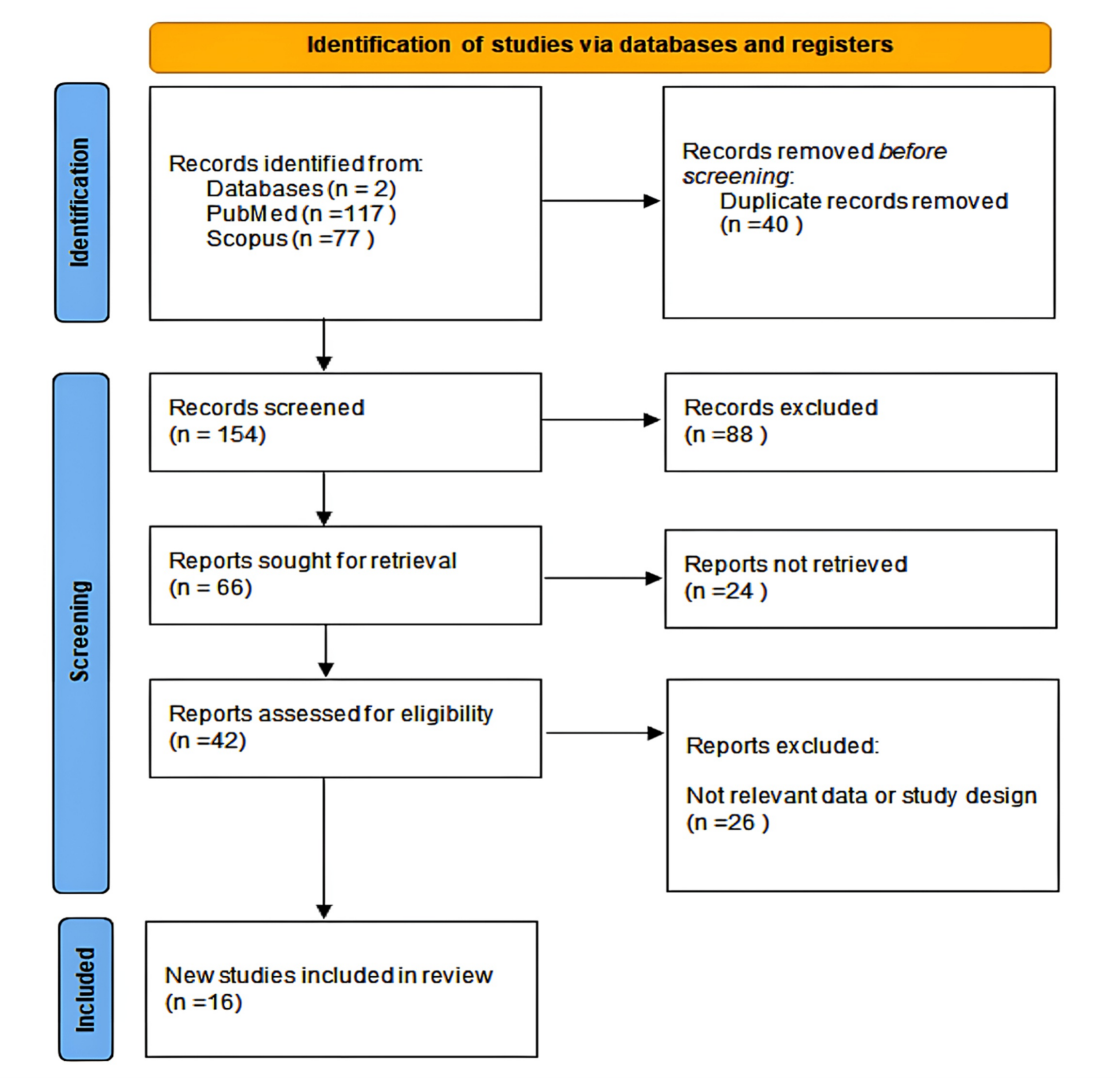
Preferred Reporting Items for Systematic Reviews and Meta-Analyses (PRISMA) flowchart of the included studies.

The Preferred Reporting Items for Systematic Reviews and Meta-Analyses (PRISMA) checklist, Figure 1 below [4], was used in conducting the systematic review. Therefore, available evidence related to the etiology, diagnosis, and treatment of Garcin syndrome was synthesized. The literature search was extensive, with a strict selection process for the inclusion of studies.

Search Strategy

A systematic search of major electronic databases was conducted: PubMed and Scopus. The following were the search terms: (Garcin syndrome) AND ("etiology" OR "diagnosis" OR "treatment" OR "management"). These terms were stringently selected in order to capture those studies that specifically address the question of the condition's etiology, diagnosis, and methods of treatment. The search strategy was intended to be broad enough to include all relevant publications without restricting the date of publication to ensure a comprehensive review of the literature.

Inclusion and Exclusion Criteria

The inclusion criterion was studied by discussing Garcin syndrome exclusively or any of its aspects regarding etiology, diagnosis, management, or treatment. Included studies should be case reports, case series, and observations. For this review, only articles in the English language should be considered. Studies not pertaining to Garcin syndrome and non-English language studies, non-peer-reviewed articles, abstracts, editorials, and those dealing with animals were excluded.

Study Selection

Database search resulted in a large output of articles. After duplicates were removed, the remaining studies went through a process of title and abstract screening. Studies satisfying the mentioned inclusion criteria for abstracts and titles underwent full-text evaluation. The eligibility of every study was evaluated by two independent reviewers. An independent third reviewer settled disagreements in the reviewers' opinions. Study selection was handled and supported by Rayyan (Cambridge, MA, USA), a web-based systematic review tool. Rayyan allowed the reviewers to collaborate efficiently, making the screening process really fast and smooth.

Data Extraction

A standardized form was used for data extraction, which included all the information from each included study. These key data points included the characteristics of the study: author, year of publication, and country of origin. The demographic data of the patients, inclusive but not limited to age, sex, and relevant clinical history, are recorded. Information regarding each case about the involvement of cranial nerves, underlying etiology, diagnostic modalities such as various imaging techniques, and biopsy reports was noted. The treatment modalities adopted, be it surgical or radiotherapy, or chemotherapy, were recorded. The data within the case files regarding the outcomes of the response to treatment, duration of follow-up, and the final status of the patient were retrieved. These data, thus extracted, were reviewed for completeness and accuracy before the synthesis.

Quality Assessment

The CARE Guidelines, Case Report [5], were used to evaluate the quality of the case reports included; this is a reporting guideline developed with the purpose of ensuring sufficient and transparent reporting of clinical cases. The title clarity and abstract of each case report were evaluated for completeness of the patient's information, completeness of clinical findings, diagnostic assessment, and therapeutic intervention. Follow-up and outcomes were also sought and reviewed in detail, and relevance and context, as provided in the discussion sections, were tabulated accordingly (Table 1) [6-21]. This approach ensured that only case reports with good documentation and of high quality could be included in the review for a reliable basis of synthesis of findings.

**Table 1:** Quality Assessment of Case Reports on Garcin Syndrome Using CARE Guidelines

Study ID	First Author	Title/Abstract	Patient Information	Clinical Findings	Diagnostic Assessment	Therapeutic Interventions	Follow-up/Outcomes	Discussion/Conclusions	Overall Quality
1	Tabari et al. [6].	Clear	Comprehensive	Detailed	Thorough	Well-documented	Well-documented	Relevant	High
2	Casolla et al. [7]	Clear	Comprehensive	Detailed	Thorough	Well-documented	Comprehensive	Relevant	High
3	Hammond et al. [8]	Clear	Comprehensive	Detailed	Thorough	Well-documented	Adequate	Relevant	High
4	Yadav et al. [9]	Clear	Comprehensive	Detailed	Thorough	Well-documented	Well-documented	Relevant	High
5	Hanse et al. [10]	Clear	Comprehensive	Detailed	Thorough	Well-documented	Comprehensive	Relevant	High
6	Bibas-Bonet et al. [11]	Clear	Comprehensive	Detailed	Thorough	Well-documented	Comprehensive	Relevant	High
7	Alapatt et al. [12]	Clear	Comprehensive	Detailed	Thorough	Adequate	Limited	Adequate	Moderate
8	Abe et al. [13]	Clear	Comprehensive	Detailed	Thorough	Adequate	Limited	Adequate	Moderate
9	Kaya et al. [14]	Clear	Comprehensive	Detailed	Thorough	Well-documented	Adequate	Relevant	High
10	Terasaki et al. [15]	Clear	Comprehensive	Detailed	Thorough	Well-documented	Well-documented	Relevant	High
11	McMenemey et al. [16]	Clear	Comprehensive	Detailed	Adequate	Adequate	Limited	Adequate	Moderate
12	Mubaidin et al. [17]	Clear	Comprehensive	Detailed	Thorough	Limited	Limited	Adequate	Moderate
13	Xia et al. [18]	Clear	Comprehensive	Detailed	Thorough	Well-documented	Comprehensive	Relevant	High
14	Fukai et al. [19]	Clear	Comprehensive	Detailed	Thorough	Well-documented	Limited	Relevant	High
15	Fujimoto et al. [20]	Clear	Comprehensive	Detailed	Thorough	Well-documented	Well-documented	Relevant	High
16	Takahashi et al. [21]	Clear	Comprehensive	Detailed	Thorough	Well-documented	Well-documented	Relevant	High

Data Synthesis

Given the heterogeneity of the included studies, especially the variability in the etiologies and different treatment approaches, a narrative synthesis was considered most appropriate. The findings were categorized into thematic categories related to the main objectives of the review: etiology, diagnosis, and treatment of Garcin syndrome. This narrative synthesis aimed to provide a comprehensive overview of the understanding of Garcin syndrome available up to this time, find patterns and challenges, and identify gaps in the literature.

Results

This systematic review presented 16 case reports on Garcin syndrome that cover broad etiologies, clinical presentations, and outcomes. Case reports were from several countries around the world, including Iran, Italy, the United Kingdom, India, the Netherlands, Argentina, Japan, Jordan, and China, signifying the worldwide presence of this condition. Years of publication ranged from 1965 through 2023, showing views of the syndrome both historically and in more contemporary times, as seen in Table 2 [6-21].

**Table 2:** Summary of Clinical Features, Diagnostic Findings, and Outcomes in Garcin Syndrome Cases CN: Cranial Nerves, CSF: Cerebrospinal Fluid, CT: Computed Tomography, DLBCL: Diffuse Large B-cell Lymphoma, FNAC: Fine Needle Aspiration Cytology, MRI: Magnetic Resonance Imaging, MRV: Magnetic Resonance Venography, NHL: Non-Hodgkin’s Lymphoma, PGACC: Parotid Gland Adenoid Cystic Carcinoma, R-CHOP-21: Rituximab, Cyclophosphamide, Doxorubicin, Vincristine, Prednisone (chemotherapy regimen).

Study ID	Author(s)	Country	Year	Sample Size	Patient Demographics	Cranial Nerves Involved	Etiology	Lesion Side	Associated Conditions	Imaging Techniques Used	Key Imaging Findings	Other Diagnostic Tests	Type of Treatment	Duration of Treatment	Response to Treatment	Follow-Up Duration	Outcome	Functional Status
1	Tabari et al. [6]	Iran	2023	1	54-year-old male	CN IX (Glossopharyngeal), CN X (Vagus), CN XII (Hypoglossal), possible involvement of CN V2 (Trigeminal)	Mucormycosis-induced skull base osteomyelitis	Right	None reported	CT scan, MRI, MRV	Destructive lesion with sequestration at the skull base, involving clivus, occiput, greater wing, and pterygoid process of the sphenoid. Heterogeneous enhancement on MRI indicating skull base osteomyelitis with possible neoplastic infiltration.	Histopathology (confirmed mucormycosis)	Endoscopic endonasal skull base debridement and biopsy	38 days of Amphotericin B post-surgery	Patient achieved physical stability post-surgery but later succumbed to complications	30 days	Death due to complications	Not reported
2	Casolla et al. [7]	Italy	2018	1	69-year-old male	CN V (Trigeminal), CN VI (Abducens), CN VII (Facial), CN VIII (Vestibulocochlear), CN IX (Glossopharyngeal), CN X (Vagus), CN XI (Accessory), CN XII (Hypoglossal)	Diffuse large B-cell lymphoma (DLBCL)	Left	None reported	MRI, CT, Lumbar puncture	MRI revealed pathological tissue spreading from oropharynx to pontocerebellar angle, compressing the root entry zone of lower cranial nerves. Erosion of temporal, occipital (clivus), and sphenoid bones. CT showed erosion of clivus and petrous ridge.	Biopsy via Hartel’s route confirmed DLBCL diagnosis	Polichemotherapy (R-CHOP-21) with high dose methotrexate, intrathecal therapy	6 cycles of chemotherapy	Significant reduction of mass volume and contrast enhancement, complete remission at 4-year follow-up	4 years	Complete remission at 4-year follow-up	Complete remission
3	Hammond et al. [8]	United Kingdom	2011	1	80-year-old male	CN V (Trigeminal), CN VI (Abducens), CN VII (Facial), CN VIII (Vestibulocochlear), CN IX (Glossopharyngeal), CN X (Vagus), CN XI (Accessory), CN XII (Hypoglossal)	Non-Hodgkin’s Lymphoma	Left	None reported	MRI, Orthopantomogram	Extra-axial enhancing lesion enveloping the hemi-mandible at the level of the left inferior alveolar nerve, extending from the left parotid mass to the skull base. Erosion of cortex and mandible. Dumbbell-shaped lesion in the cerebellopontine angle.	Incisional biopsy confirmed NHL	Primary chemo-radiotherapy	Not specified	Significant reduction in lesion size, but details on long-term outcome not provided	Not specified	Not specified	Not reported
4	Yadav et al. [9]	India	2021	1	33-year-old male	CN I (Olfactory), CN II (Optic), CN III (Oculomotor), CN IV (Trochlear), CN V (Trigeminal), CN VI (Abducens), CN VII (Facial), CN VIII (Vestibulocochlear), CN IX (Glossopharyngeal), CN X (Vagus), CN XI (Accessory), CN XII (Hypoglossal)	Invasive mucormycosis	Left	Type 1 Diabetes Mellitus His blood sugar levels were uncontrolled (300 mg/ml)	CT scan, Nasal Endoscopy	Soft tissue density filling anterior and posterior ethmoids and sphenoid sinuses, erosion of the medial orbital wall reaching the orbital apex and compressing the optic nerve.	Biopsy confirmed mucormycosis	Intravenous insulin, Liposomal Amphotericin B, Surgical debridement	6 weeks of Amphotericin B	Significant improvement in general condition, partial recovery of cranial nerve functions	12 weeks	Improved	Partial recovery of CN functions
5	Hanse et al. [10]	Netherlands	2003	1	47-year-old male	CN I (Olfactory), CN II (Optic), CN III (Oculomotor), CN IV (Trochlear), CN V (Trigeminal), CN VI (Abducens), CN VII (Facial), CN VIII (Vestibulocochlear), CN IX (Glossopharyngeal), CN X (Vagus), CN XII (Hypoglossal)	Rhinocerebral mucormycosis	Right	Newly diagnosed diabetes mellitus	MRI, Cerebrospinal fluid analysis	MRI showed slight enhancement of leptomeninges of the brainstem, congested nasal sinuses, and a lesion in the right orbit with enhancement of leptomeninges.	Maxillary tap, Microscopic examination confirmed mucorales hyphae (Rhizopus oryzae)	Amphotericin B, Vancomycin, Ethmoidectomy, Partial resection of orbit	4 weeks of Amphotericin B (continued with liposomal form due to renal insufficiency)	Patient in good clinical condition two years post-treatment	2 years	Good clinical condition	Renal insufficiency developed during treatment
6	Bibas-Bonet et al. [11]	Argentina	2003	1	8-year-old female	CN V (Trigeminal), CN VI (Abducens), CN VII (Facial), CN VIII (Vestibulocochlear), CN IX (Glossopharyngeal), CN X (Vagus), CN XI (Accessory), CN XII (Hypoglossal)	Giant cell tumor of the skull base	Right	None reported	CT scan, MRI	Lytic lesions in the right temporal petrous portion, destructive mass involving the right greater wing of the sphenoid bone and temporal petrous apex, compression of brainstem but no invasion, no hydrocephalus	Biopsy confirmed giant cell tumor	Radiotherapy (50 Gy of cobalt-60)	5-week course	Clinical improvement, no evidence of tumor growth at 8-year follow-up	8 years	Well but with functional sequelae	Right-sided deafness, swallowing difficulties, facial paresis, mild tongue wasting
7	Alapatt et al. [12]	India	2007	1	45-year-old male	CN II (Optic), CN III (Oculomotor), CN IV (Trochlear), CN V (Trigeminal), CN VI (Abducens), CN VII (Facial), CN IX (Glossopharyngeal), CN X (Vagus), CN XII (Hypoglossal)	Poorly differentiated carcinoma of nasopharynx	Right	None reported	MRI	Ill-defined enhancing lesion in the right cavernous sinus, Meckel’s cave, with extension to the right orbit, superior orbital fissure, posterior fossa, and cheek	Fine needle aspiration cytology (FNAC) confirmed poorly differentiated carcinoma.	None specified, patient presented for diagnosis after disease progression	Not specified	Not specified	Not specified	Death	Progression of cranial nerve palsies with proptosis, pain, and numbness on the right side of the face
8	Abe et al. [13]	Japan	1988	1	71-year-old female	CN II (Optic), CN III (Oculomotor), CN IV (Trochlear), CN V (Trigeminal), CN VI (Abducens), CN VIII (Vestibulocochlear), CN IX (Glossopharyngeal), CN X (Vagus), CN XII (Hypoglossal)	Nasopharyngeal carcinoma invading the skull base	Left	Hypopituitarism	MRI	Tumor invasion from the Eustachian tube to the foramen magnum, sella turcica, and pituitary gland. The tumor pushed the pituitary gland forward and invaded it later, causing hypopituitarism.	Brain biopsy confirmed poorly differentiated squamous cell carcinoma	None specified	Not specified	Not specified	Not specified	Tumor invasion led to hypopituitarism	None reported
9	Kaya et al. [14]	Japan	1995	1	15-year-old female	CN II (Optic), CN III (Oculomotor), CN IV (Trochlear), CN V (Trigeminal), CN VI (Abducens), CN VII (Facial), CN VIII (Vestibulocochlear), CN IX (Glossopharyngeal), CN X (Vagus), CN XII (Hypoglossal)	Acute non-lymphocytic leukemia complicated by Garcin’s syndrome	Right	None reported	CT scan, MRI	Mass infiltrating the skull base with extensive cranial nerve involvement, affecting the middle and posterior cranial fossae.	Bone marrow biopsy confirmed acute non-lymphocytic leukemia	Chemotherapy (specific regimen not detailed)	Not specified	Partial improvement of symptoms, but significant residual deficits	Not specified	Residual deficits in cranial nerve function	None reported
10	Terasaki et al. [15]	Japan	2021	1	58-year-old female	CN V (Trigeminal), CN VI (Abducens), CN VII (Facial), CN VIII (Vestibulocochlear), CN IX (Glossopharyngeal), CN X (Vagus), CN XII (Hypoglossal)	Meningeal carcinomatosis from gastric cancer	Left	None reported	CT scan, MRI, CSF cytology	Initial CT and MRI were normal; later gadolinium-enhanced MRI revealed abnormal dense staining along cranial nerves; CSF cytology confirmed signet ring cells consistent with gastric cancer	CSF cytology confirmed meningeal carcinomatosis with signet ring cells	Whole-brain radiation therapy, planned intrathecal chemotherapy	4 sessions of radiation therapy	Patient died four days after diagnosis despite treatment	49 days	Death	Rapid progression of cranial nerve palsy
11	McMenemey et al. [16]	United Kingdom	1965	1	51-year-old male.	CN V (Trigeminal), CN VI (Abducens), CN VII (Facial), CN VIII (Vestibulocochlear), CN IX (Glossopharyngeal), CN X (Vagus), CN XII (Hypoglossal)	Stern-Garcin syndrome post-traffic accident	Left	Depression	Not specified	Not specified	Postmortem examination revealed thalamic degeneration	None specified	Not specified	Patient died 12 weeks post-accident due to terminal uremia	12 weeks	Death	Severe memory loss, myoclonic twitching
12	Mubaidin et al. [17]	Jordan	1990	1	50-year-old male	CN I (Olfactory), CN II (Optic), CN III (Oculomotor), CN IV (Trochlear), CN V (Trigeminal), CN VI (Abducens), CN VII (Facial), CN VIII (Vestibulocochlear), CN IX (Glossopharyngeal), CN X (Vagus), CN XI (Accessory), CN XII (Hypoglossal)	Renal cell carcinoma with metastasis to the skull base	Right	None reported	CT scan, Skull radiography	CT scan showed a large osteolytic soft tissue tumor with marked enhancement destroying the tip of the petrous, sphenoid, and ethmoid bones, with intracranial extension to the right temporal lobe, cerebellar hemisphere, pons, and midbrain.	Necropsy confirmed renal cell carcinoma with skull base metastases	None (patient declined surgery and further treatment)	Not applicable	Patient died shortly after diagnosis due to disease progression	Not reported	Death	Severe cranial nerve palsies leading to progressive functional decline
13	Xia et al. [18]	China	2017	1	59-year-old female	CN V (Trigeminal), CN VII (Facial), CN VIII (Vestibulocochlear), CN IX (Glossopharyngeal), CN X (Vagus), CN XI (Accessory), CN XII (Hypoglossal)	Parotid gland adenoid cystic carcinoma (PGACC)	Left	None reported	MRI	Extensive abnormal signal in the left parapharyngeal space, hard palate, root of the tongue, and parotid gland with strong homogeneous enhancement on gadolinium-enhanced MRI.	Parotid gland biopsy confirmed PGACC with perineural invasion	Three rounds of local-field palliative radiotherapy	Several months	Patient died several months after treatment due to disease progression	Not specified	Death	Progressive worsening of cranial nerve palsies
14	Fukai et al. [19]	Japan	2018	1	76-year-old female	CN IV (Trochlear), CN V1 (Trigeminal - Ophthalmic branch), CN VI (Abducens)	Sphenoid bone metastasis from lung cancer (pleomorphic carcinoma)	Left	None reported	CT scan, MRI	Bone destruction at the left sphenoid sinus on CT; contrast-enhanced tumor in the sphenoid sinus on gadolinium-enhanced MRI	None reported	Palliative radiotherapy (5 Gy × 4 sessions), zoledronic acid	4 sessions of radiation therapy	No neurological improvement, patient’s condition worsened	5 months	Death	Worsening of general condition, no improvement in neurological symptoms
15	Fujimoto et al. [20]	Japan	2002	1	71-year-old male	CN V (Trigeminal), CN VI (Abducens), CN VII (Facial), CN VIII (Vestibulocochlear), CN IX (Glossopharyngeal), CN X (Vagus), CN XII (Hypoglossal)	Paraneoplastic sensory neuronopathy with anti-Hu antibodies from small-cell lung cancer	Left	None reported	MRI, CSF analysis	No tumor metastasis or invasion seen in MRI; pathological examination revealed marked neuronal loss, gliosis, and perivascular lymphocytic infiltration in brainstem	Sural nerve biopsy, postmortem examination	Systemic chemotherapy (details not specified)	13 months	No improvement, patient died of respiratory failure	13 months	Death	Multiple cranial nerve palsies, respiratory failure
16	Takahashi et al. [21]	Japan	1982	1	6-year-old male	CN III (Oculomotor), CN IV (Trochlear), CN V (Trigeminal), CN VI (Abducens), CN VII (Facial), CN VIII (Vestibulocochlear), CN IX (Glossopharyngeal), CN X (Vagus), CN XI (Accessory), CN XII (Hypoglossal)	Rhabdomyosarcoma in the head and neck	Left	None reported	Skull radiography, Carotid and vertebral angiograms	Skull radiography showed destructive lesions in the base of the skull; angiograms revealed occlusion of the left internal carotid artery and collateral circulation to the left cerebral hemisphere.	Biopsy of the tumor in the left retromandibular region confirmed rhabdomyosarcoma	Radiation therapy (5,100 rads, followed by additional 3,000 rads)	9 months	Progressive deterioration, leading to death	1 year	Death	Severe cranial nerve palsies, visual deterioration, tumor progression

Patient Demographics

The age of the patients in the reviewed cases ranged from six to 80 years. The majority were male subjects, although quite a number of the cases did involve female patients, especially in the pediatric population. All classes of individuals with varied backgrounds and medical histories were affected by this syndrome. However, the common threaded presence of underlying risk factors included diabetes mellitus, malignancy, or history of immunocompromise.

Cranial Nerve Involvement

Garcin syndrome is characterized by progressive unilateral involvement of multiple cranial nerves. Among the reviewed cases, the trigeminal nerve CN V, facial nerve CN VII, and hypoglossal nerve CN XII were the most commonly involved cranial nerves. Oculomotor nerve CN III, abducens nerve CN VI, and vestibulocochlear nerve CN VIII were also not infrequently involved nerves. Of note are those cases reporting as many as 12 cranial nerves involved, which point toward the extensive nature of the syndrome. The involvement is often unilateral but may progress to severe neurological deficits, including facial paralysis, dysphagia, and loss of ocular movements.

Etiology

Among these, the most common causes of Garcin syndrome were neoplastic and infectious in origin. The leading causes are skull base tumors, both primary and metastatic. Primary tumors included skull neoplasms such as nasopharyngeal carcinoma, parotid gland adenoid cystic carcinoma, and rhabdomyosarcoma. Metastatic lesions were commonly observed from primary sites of the lung, including but not limited to pleomorphic carcinoma, and the kidneys, like renal cell carcinoma. These were usually aggressive tumors with a tendency for local invasion of the skull base, with subsequent destruction and involvement of the cranial nerves.

The infectious etiologies have also been significant, especially in patients with an immunocompromised state. In a few cases, life-threatening fungal infection invasive mucormycosis was noted to be the cause of this. Poorly controlled diabetes mellitus was noted quite frequently in such infections, pointing toward good metabolic control for the prevention of such devastating complications.

Lesion Laterality

In all instances, laterality of the lesion was noted, with right- and left-sided lesions relatively evenly divided. In eight instances, the lesion was on the right side, while in eight other cases, the lesion was on the left side. Symptoms, in most instances, correlated to the side of the lesion since many patients often presented with unilateral cranial nerve palsies corresponding to the side of the lesion.

Imaging and Diagnostic Findings

Imaging techniques, like MRI and CT, were especially important in the diagnosis of Garcin syndrome, showing typical destructive lesions at the base of the skull, including bone erosion, tumor infiltration, and sometimes intracranial extension. MRI well evaluated soft tissue involvement and the degree of compression on the cranial nerves. For instance, cases of mucormycosis were presented as soft tissue density within the sinuses, with erosions of contiguous bony structures, while neoplastic lesions would usually result in significant erosion of the bones and a mass effect. Apart from the imaging, diagnosis also had to be confirmed on histopathology and cytology. In fact, biopsies were undertaken in the majority, especially to further delineate neoplastic from infectious causes. In the cases with malignancy, these features were supported by the pathologic diagnosis through biopsy, showing aggressive tumors such as diffuse large B-cell lymphoma (DLBCL) and poorly differentiated squamous cell carcinoma (SCC), a common finding in perineural invasion (PNI) [6-21].

Treatment Modalities

These treatment modalities were quite different since they are linked to the underlying etiology of Garcin syndrome. In the case of mucormycosis, aggressive surgery for debridement together with administration of antifungals, preferably amphotericin B, is the standard modality. Despite these efforts, prognosis in such cases often was poor owing to the advanced disease stage at diagnosis and the rapid spread of infection.

The neoplastic causes responded to chemotherapy and radiotherapy. Patients with DLBCL, for example, generally received R-CHOP-21 (rituximab, cyclophosphamide, doxorubicin, vincristine, prednisone) chemotherapy supplemented with high-dose methotrexate and intrathecal therapy, which reduced tumor size notably, while some achieved complete remission. In general, response to treatment was variable in most patients because the disease progressed in quite a few of them despite aggressive treatment management. Indication for surgical intervention was less common in neoplastic cases because of the difficult site of lesions and the risks from skull base surgery.

Palliative care was an important modality of management, particularly in those with extensive cranial nerve involvement or metastatic disease. Meningeal carcinomatosis was treated with whole-brain radiation therapy. In most instances, however, this resulted in a rather poor response, with most patients dying from the disease within several months of establishing the diagnosis.

Outcomes

By nature, Garcin syndrome tends to run a natural history of poor patient outcomes, reflecting the aggressive nature of the underlying etiologies. Indeed, the majority of patients in the reviewed 16 cases either suffered significant residual neurological deficits or died from the disease. For example, in those cases involving invasive mucormycosis, despite the use of aggressive treatments, many patients died from the lethal infectious disease process in the immunocompromised host.

The prognosis was equally grim in neoplastic etiology, particularly with metastatic diseases. The survival times were from mere weeks from diagnosis to several years, especially in those who responded rather well to the chemotherapy. However, long survivors were few, and the quality of life was badly compromised by constant cranial nerve palsies and other sequelae.

Complications

Complications were almost the rule, related both to the basic disease and to the therapies employed. Renal insufficiency was a complication to be anticipated; prolonged antifungal treatment, especially with amphotericin B, was the cause of renal failure in these patients. Radiation therapy resulted in functional sequelae: deafness, facial paresis, dysphagia, etc.

Discussion

Garcin syndrome is a very rare and complex neurological disorder. This may include progressive, unilateral involvement of multiple cranial nerves sans intracranial hypertension, where the brain involvement is minimal, even though cranial nerve involvement may be extensive. Probably, this is the most puzzling thing in this syndrome-the diagnosis is complicated, and clinicians are forced to think of a wide range of differential diagnoses, from tumors to infections and autoimmune conditions [22]. The present systematic review details an overview of Garcin syndrome by using various causes, clinical manifestations, and results that can occur with this rare and complicated disease. This review includes 16 case reports from different continents and over several decades that detail the worldwide importance and outline clinically significant obstacles presented by Garcin syndrome.

The cases reviewed focus on the neoplastic and infectious causes of Garcin syndrome, reflecting its strong association with aggressive underlying pathologies. The leading causes were skull base tumors, which were both malignant and metastatic. Hammond et al. [8], for instance, described a case of Garcin syndrome secondary to non-Hodgkin's lymphoma where there was extensive cranial nerve involvement due to the aggressive invasion of the tumor at the base of the skull. Similarly, Casolla et al. (2018) [7] also described the case of diffuse large B-cell lymphoma that has huge destruction capability at the skull base, leading to severe and progressive neurological deficits. Regarding infectious etiology, especially invasive mucormycosis, this played an important role, particularly in the immunocompromised patient population. Yadav et al. (2021) [9] and Hanse et al. (2003) [10] have variously reported that poorly controlled diabetes mellitus predisposed the patients to mucormycosis, which progressed with severe cranial nerve palsies as an extension into the skull base. These are just cases highlighting how the management of underlying conditions like diabetes is important in preventing such devastating complications.

The case report by Massey et al. [23] provides insight into an unusual etiology of Garcin syndrome, as it was due to an adenoid cystic carcinoma. This infrequent, slow-growing malignancy usually originates in the salivary glands and tends to perineural invasion, thus giving rise to a widespread involvement of cranial nerves. In this case, the patient presented with progressive cranial nerve palsies, starting with numbness of the face and ocular symptoms, which later included nearly all one-sided cranial nerves. Likewise, despite multiple imaging and high suspicion for the diagnosis, the disease was diagnosed late in this case. This case underlines the diagnostic difficulties in adenoid cystic carcinoma and implies, as always, the importance of early diagnosis and treatment to reduce the level of impairment.

The case report by Roubeau et al. (2012) [24] gives a detailed history of Garcin syndrome due to PNI caused by SCC. The above case is a delineation of PNI as a mechanism of cutaneous malignancies presenting themselves as an extensive cranial nerve involvement leading to Garcin syndrome. In this case, progressive cranial nerve palsies occurred as a result of PNI from a previously treated SCC of the temporal scalp. The carcinoma, which initially presented and was treated, recurred and invaded cranial nerves. It points out the diagnostic difficulties posed by PNI and requires extra clinical effort to raise suspicion, thus often requiring frequent imaging studies in patients with SCC recurrence.

The interesting case report by Kono (1970) [25] details a rather unusual etiology of Garcin syndrome, wherein the syndrome was secondary to a primary carcinoma of the middle ear. This case is instructive inasmuch as it constitutes one of the few on record that have directly implicated a middle ear tumor in the development of Garcin syndrome. There was a unilateral involvement of nearly all the cranial nerves on the right side, which is in keeping with the clinical definition of Garcin syndrome. The extensive local invasion by the tumor led to severe damage to multiple cranial nerves. The case thus underscores the importance of early recognition and intervention in Garcin syndrome, particularly when it is secondary to such rare and aggressive tumors as middle ear carcinoma.

Garcin syndrome is characterized by the involvement of multiple cranial nerves unilaterally, often without signs of increased intracranial pressure. Extensive involvement of cranial nerves was a feature in all reviewed cases, ranging from some patients who had up to 12 nerves affected. For example, in the case described by Bibas-Bonet et al. (2003) [11], a giant cell tumor of the skull base involved all major cranial nerves on one side with profound neurological deficits, including facial paralysis, dysphagia and loss of ocular movements. The complexity of Garcin syndrome is particularly evident when it presents as the initial manifestation of underlying malignancies or severe infections. For instance, Kubota et al. (2012) [2] presented the case of a rare condition wherein Garcin syndrome was the initial presentation of metastatic adenocarcinoma of the prostate. This case, therefore, underlines the consideration of skull base metastases in patients presenting with unexplained and progressive cranial neuropathies, even if the primary tumors, such as those originating from the prostate, are not normally associated with cranial neuropathies. Similarly, a case of Garcin syndrome associated with metastatic colorectal carcinoma was demonstrated by Bedi et al. (2004) [1], further illustrating the various list of causes for the syndrome. Another case reported in 2022 by Darazam et al. [3] highlights further challenges in the course of the disease for immunocompromised patients, who carry an increased risk of developing aggressive neoplasms that complicate Garcin syndrome. These patients usually have a blunted response to treatment, which further contributes to their poor outcomes. Thus, early recognition of Garcin syndrome coupled with prompt and aggressive management of the underlying cause is critical to improve the prognosis of such patients, although overall outcomes remain challenging. The case report by Yang et al. (2016) [26] describes the diagnostic challenges offered by Garcin syndrome in cases secondary to rhinocerebral mucormycosis (RCM). Mucormycosis is an infrequent but highly aggressive mycosis, with a catastrophic course in immunocompromised subjects. The inaugural misdiagnosis of tuberculous meningitis underlines the diagnostic challenge concerning RCM in Garcin syndrome. This case stresses the need for sharp clinical awareness of RCM as a cause of Garcin syndrome and the need for early diagnosis to improve outcomes (Table 3).

**Table 3:** Comprehensive Summary of Etiologies, Cranial Nerve Involvement, Management Plans, and Outcomes in Garcin Syndrome Cases. CN: Cranial Nerves, DLBCL: Diffuse Large B-cell Lymphoma, FNAC: Fine Needle Aspiration Cytology, MRI: Magnetic Resonance Imaging, RCM: Rhinocerebral Mucormycosis, R-CHOP-21: Rituximab, Cyclophosphamide, Doxorubicin, Vincristine, Prednisone (chemotherapy regimen), SCC: Squamous Cell Carcinoma, WBRT: Whole Brain Radiation Therapy.

Etiology	Cranial Nerves Involved	Management Plan	Outcome
Mucormycosis-induced skull base osteomyelitis	CN IX (Glossopharyngeal), CN X (Vagus), CN XII (Hypoglossal), possible CN V2 (Trigeminal)	Surgical: Endoscopic skull base debridement and biopsy. Medical: Amphotericin B for 38 days post-surgery. Early aggressive surgical intervention and antifungal therapy were critical.	Patient achieved physical stability post-surgery but later succumbed to complications.
Diffuse large B-cell lymphoma (DLBCL)	CN V (Trigeminal), CN VI (Abducens), CN VII (Facial), CN VIII (Vestibulocochlear), CN IX (Glossopharyngeal), others	Medical: R-CHOP-21 chemotherapy, high-dose methotrexate, intrathecal therapy. Surgical: Biopsy via Hartel’s route. Aggressive chemotherapy was crucial for tumor reduction and remission.	Complete remission at 4-year follow-up.
Non-Hodgkin’s lymphoma	CN V (Trigeminal), CN VI (Abducens), CN VII (Facial), CN VIII (Vestibulocochlear), CN IX (Glossopharyngeal), others	Medical: Chemotherapy and radiotherapy. Surgical: Incisional biopsy. The focus was on reducing tumor size and controlling disease progression.	Reduction in lesion size, long-term outcome not provided.
Invasive mucormycosis	All cranial nerves, including CN I (Olfactory) and CN XII (Hypoglossal)	Surgical: Nasal endoscopy and debridement. Medical: Liposomal Amphotericin B for 6 weeks, intravenous insulin. Combined surgical and antifungal therapy improved general condition.	Partial recovery of cranial nerve functions.
Rhinocerebral mucormycosis	All cranial nerves, including CN I (Olfactory) and CN XII (Hypoglossal)	Surgical: Ethmoidectomy, partial resection of orbit. Medical: Amphotericin B, later liposomal Amphotericin B due to renal insufficiency, Vancomycin. Management focused on fungal control and tissue preservation.	Good condition at 2-year follow-up.
Giant cell tumor of the skull base	CN V (Trigeminal), CN VI (Abducens), CN VII (Facial), CN VIII (Vestibulocochlear), CN IX (Glossopharyngeal), others	Surgical: Biopsy to confirm diagnosis. Medical: Radiotherapy (50 Gy of cobalt-60). Early intervention controlled tumor growth.	No evidence of tumor growth at 8-year follow-up, with functional sequelae.
Poorly differentiated nasopharyngeal carcinoma	CN II (Optic), CN III (Oculomotor), CN IV (Trochlear), CN V (Trigeminal), CN VI (Abducens), CN VII (Facial), others	Diagnostic: MRI and FNAC. Management: No specific treatment was initiated as the patient presented after significant disease progression.	Death from disease progression.
Adenoid cystic carcinoma (cylindroma)	Nearly all cranial nerves on one side	Surgical: None mentioned (late-stage diagnosis). Diagnostic: MRI, biopsy to confirm diagnosis. Management emphasized early detection, but late diagnosis led to severe progression.	Death due to late-stage diagnosis.
Squamous cell carcinoma (SCC) with perineural invasion	CN V (Trigeminal), CN VI (Abducens), CN VII (Facial), CN VIII (Vestibulocochlear), CN IX (Glossopharyngeal), others	Medical: radiotherapy. Surgical: No specific surgical intervention was detailed; focus on management through radiotherapy.	Death due to disease recurrence and progression.
Primary carcinoma of the middle ear	Nearly all cranial nerves on the right side	Medical: Radiotherapy. Diagnostic: Extensive imaging to confirm diagnosis. The focus was on managing the tumor and relieving symptoms, though prognosis remained poor due to late-stage detection.	Death from disease progression.
Metastatic adenocarcinoma of the prostate	Multiple cranial nerves, specific nerves not listed	Diagnostic: MRI to detect metastases. Medical: Palliative care and possibly radiotherapy. The case emphasized the importance of considering skull base metastases in unexplained cranial nerve palsies.	Rapid disease progression and death.
Metastatic colorectal carcinoma	CN V (Trigeminal), CN VII (Facial), others	Medical: Palliative radiotherapy. Diagnostic: MRI to detect metastases. The case highlighted the challenges of managing Garcin syndrome secondary to aggressive metastatic diseases.	Poor prognosis and death.
Parapharyngeal diffuse large B-cell lymphoma (AIDS)	Multiple cranial nerves, specific nerves not listed	Medical: Aggressive chemotherapy. Diagnostic: Imaging and histopathology to confirm diagnosis. The patient’s immunocompromised state complicated the management, underscoring the need for early intervention.	Poor prognosis and death.
Parotid gland adenoid cystic carcinoma	CN V (Trigeminal), CN VII (Facial), CN VIII (Vestibulocochlear), CN IX (Glossopharyngeal), others	Surgical: Parotid gland biopsy. Medical: Palliative radiotherapy. Management focused on palliative care due to extensive cranial nerve involvement and disease progression.	Death due to disease progression.
Paraneoplastic sensory neuronopathy from small-cell lung cancer	CN V (Trigeminal), CN VI (Abducens), CN VII (Facial), CN VIII (Vestibulocochlear), CN IX (Glossopharyngeal), others	Medical: Systemic chemotherapy. Diagnostic: Sural nerve biopsy, postmortem examination. Aggressive chemotherapy aimed at controlling cancer but was ultimately unsuccessful.	Death due to respiratory failure after progressive cranial nerve involvement.
Rhabdomyosarcoma in the head and neck	CN III (Oculomotor), CN IV (Trochlear), CN V (Trigeminal), CN VI (Abducens), CN VII (Facial), CN VIII (Vestibulocochlear), others	Surgical: Biopsy of the tumor in the left retromandibular region. Medical: Radiation therapy (5100 rads followed by additional 3000 rads). Early and aggressive radiation therapy was crucial in attempting to control tumor progression.	Death due to tumor progression despite aggressive therapy.
Rhinocerebral mucormycosis	Multiple cranial nerves, specific nerves not listed	Medical: Amphotericin B therapy, anti-tuberculous therapy initially. Diagnostic: Biopsy confirmed RCM. Highlighted the diagnostic challenges and importance of early intervention in managing mucormycosis in immunocompromised patients.	Death due to rapid progression of RCM.
Metastatic breast cancer	CN V (Trigeminal), CN VII (Facial), others	Medical: Hippocampal-sparing WBRT. Surgical: None specified. This case highlighted a novel approach to radiation therapy aimed at preserving cognitive function while effectively managing cranial nerve involvement.	Symptom resolution, but long-term prognosis not provided

A case reported by Tadokoro et al. (2020) [27] deposes the diagnostic challenge in distinguishing Garcin syndrome from other associated conditions in neurology, among them myasthenia gravis (MG). The case of a 59-year-old male presenting with Garcin syndrome symptoms, characterized by multiple cranial nerve palsies, further testing turned out positive for MG through positive anti-acetylcholine receptor antibodies, including a positive edrophonium test. The patient received oral prednisolone and intravenous immunoglobulin, to which he showed an impressive response. This case underlines that MG and other neuromuscular disorders should be considered in the differential diagnosis of Garcin syndrome, especially when typical tumor-related findings are absent. This accurate diagnosis dramatically alters the management approach from oncologic to immunomodulatory therapy.

Imaging played a very important role in the diagnosis of Garcin syndrome in all cases. MRI and CT scans provided the necessary detail regarding the extent of bony destruction, soft tissue involvement, and cranial nerve compression. For instance, MRI findings by Xia et al. (2017) [18] showed extensive involvement of the parapharyngeal space extending into the hard palate and parotid gland, which were crucial for the diagnosis of the underlying adenoid cystic carcinoma. Similarly, Takahashi et al. (1982) [21] used skull radiography and angiography in the detection of destructive lesions and vascular involvement in one case of rhabdomyosarcoma. With all advances in imaging modalities, Garcin syndrome remains difficult to diagnose on account of the insidious mode of onset and wide differential diagnosis for cranial nerve palsies. This is further complicated when the syndrome presents as an initial manifestation of an underlying malignancy, as in the case described by Mubaidin et al. (1990) [17], with metastasis to the skull base from renal cell carcinoma presenting at the outset as Garcin syndrome.

In such situations, the modality of treatment was usually dependent on the underlying cause. For example, the approach to mucormycosis was the aggressive surgical debridement accompanied by antifungal medication, with a very poor prognosis, while neoplastic causes were managed mainly with chemotherapy and radiotherapy. Tabari et al. (2023) [6] reported a case that showed that despite early surgical intervention and antifungal medication, the patient died from complications related to the disease. For instance, Casolla et al. (2018) [7] describe a case of patients with DLBCL, treated with chemotherapy R-CHOP, who demonstrated marked tumor reductions; the overall prognosis for neoplastic causes was variably poor and generally poor in cases with metastasis. For example, Terasaki et al. (2021) [15] described a case in which meningeal carcinomatosis due to gastric cancer led to a fulminant course with a lethal outcome within 49 days despite treatments. In most cases, particularly when the disease had already been at its advanced stage with any possibility of curative treatment out of the window, the mainstay of care was supportive/palliative. This review demonstrated that long-term survival was rare, and most of their patients died or had significant morbidity, just like the case reports presented by Fujimoto et al. (2002) [20] and Fukai et al. (2018) [19].

The case report by Bonzano et al. (2022) [28] highlighted a novel approach to managing Garcin syndrome in the setting of metastatic disease and underlined the possible advantage represented by advanced radiation techniques, such as hippocampal-sparing whole brain radiation therapy (WBRT). Hippocampal-sparing WBRT represents a novel approach to treating cranial nerve involvement in Garcin syndrome while minimizing cognitive side effects. This case points out the potential of hippocampal-sparing WBRT as a promising treatment option for Garcin syndrome, especially in metastatic patients.

Generally, the prognosis of Garcin syndrome remains unfavorable, especially in patients with both metastatic disease and serious infection. Despite recent advances in diagnostic and therapeutic approaches, multifocal involvement of cranial nerves carries an especially poor prognosis in the presence of an underlying malignancy. This review, therefore, emphasizes that Garcin syndrome is not only severe but usually lethal and calls for further research to find more practical diagnostic and therapeutic strategies. With already diverse etiologies and extension of cranial nerve involvement, diagnosis, and management are difficult, and the need for early intervention and multidisciplinary management is very critical.

## Conclusions

This systematic review has underlined that Garcin syndrome is a serious and often lethal condition, usually because of aggressive malignancies and invasive infections. The varied causes of Garcin syndrome, which range from skull base tumors and metastases to rarer conditions like adenoid cystic carcinoma and middle ear carcinomas, further complicate the diagnosis and management of this disease. These cases, as reviewed, also show that the prognosis for such patients is still dismal, with most patients suffering from grave morbidity or mortality despite advances in diagnostic imaging and new therapeutic strategies. Future studies should be directed at improvement in early diagnostic techniques, especially in those cases where Garcin syndrome will be the presenting feature of some occult malignancy or infection. This would be enhanced by the development of more sensitive imaging modalities and biomarker-driven diagnostics; these, in turn, would allow for earlier and more discriminate interventions. Because of the poor prognosis associated with current treatment modalities, innovative strategies coupled with a multidisciplinary approach to patient care are required if one hopes to improve survival and quality of life in individuals affected by this challenging condition.
